# Machine Learning Algorithms to Distinguish Myocardial Perfusion SPECT Polar Maps

**DOI:** 10.3389/fcvm.2021.741667

**Published:** 2021-11-11

**Authors:** Erito Marques de Souza Filho, Fernando de Amorim Fernandes, Christiane Wiefels, Lucas Nunes Dalbonio de Carvalho, Tadeu Francisco dos Santos, Alair Augusto Sarmet M. D. dos Santos, Evandro Tinoco Mesquita, Flávio Luiz Seixas, Benjamin J. W. Chow, Claudio Tinoco Mesquita, Ronaldo Altenburg Gismondi

**Affiliations:** ^1^Post-graduation in Cardiovascular Sciences, Universidade Federal Fluminense, Niterói, Rio de Janeiro, Brazil; ^2^Department of Languages and Technologies, Universidade Federal Rural do Rio de Janeiro, Rio de Janeiro, Brazil; ^3^Department of Nuclear Medicine, Hospital Universitário Antônio Pedro/EBSERH, Universidade Federal Fluminense, Rio de Janeiro, Brazil; ^4^Department of Cardiac Image, University of Ottawa Heart Institute, Ottawa, ON, Canada; ^5^Institute of Computing, Universidade Federal Fluminense, Rio de Janeiro, Brazil; ^6^Department of Nuclear Medicine, Hospital Pró-Cardíaco, Americas Serviços Medicos, Rio de Janeiro, Brazil

**Keywords:** machine learning, polar maps, myocardial perfusion imaging (MPI), coronary artery disease, random forest

## Abstract

Myocardial perfusion imaging (MPI) plays an important role in patients with suspected and documented coronary artery disease (CAD). Machine Learning (ML) algorithms have been developed for many medical applications with excellent performance. This study used ML algorithms to discern normal and abnormal gated Single Photon Emission Computed Tomography (SPECT) images. We analyzed one thousand and seven polar maps from a database of patients referred to a university hospital for clinically indicated MPI between January 2016 and December 2018. These studies were reported and evaluated by two different expert readers. The image features were extracted from a specific type of polar map segmentation based on horizontal and vertical slices. A senior expert reading was the comparator (gold standard). We used cross-validation to divide the dataset into training and testing subsets, using data augmentation in the training set, and evaluated 04 ML models. All models had accuracy >90% and area under the receiver operating characteristics curve (AUC) >0.80 except for Adaptive Boosting (AUC = 0.77), while all precision and sensitivity obtained were >96 and 92%, respectively. Random Forest had the best performance (AUC: 0.853; accuracy: 0,938; precision: 0.968; sensitivity: 0.963). ML algorithms performed very well in image classification. These models were capable of distinguishing polar maps remarkably into normal and abnormal.

## Introduction

Myocardial perfusion imaging (MPI) plays an essential role in the diagnosis and risk stratification of a patient with suspected and documented coronary artery disease (CAD) (1). Thus, accurate reporting of MPI is paramount and requires experienced professionals (2–4). Interpretation errors by health professionals can impact patient care and need to be minimized. The use of MPI is common. For instance, 61.9 studies were performed for every 1,000 Medicare beneficiaries in 2013. In Australia, there were 337 MPI studies per 100,000 people in 11 years (5). High volumes, increasing workload, and clinical demands can potentially lead to interpretation errors. Therefore, a decision support tool capable of interpreting could improve efficiency, accuracy, and costs (6).

Artificial intelligence (AI) has the potential to improve healthcare delivery. It combines mathematical models and computation, designed to emulate human intelligence (7). In particular, machine learning (ML), a subset of AI models, encompasses several methods capable of performing tasks after exposure to data (8). It has gained relevance in medicine and has transitioned from structured data to image analysis in diagnostic imaging and MPI (9).

**Figure 1:**
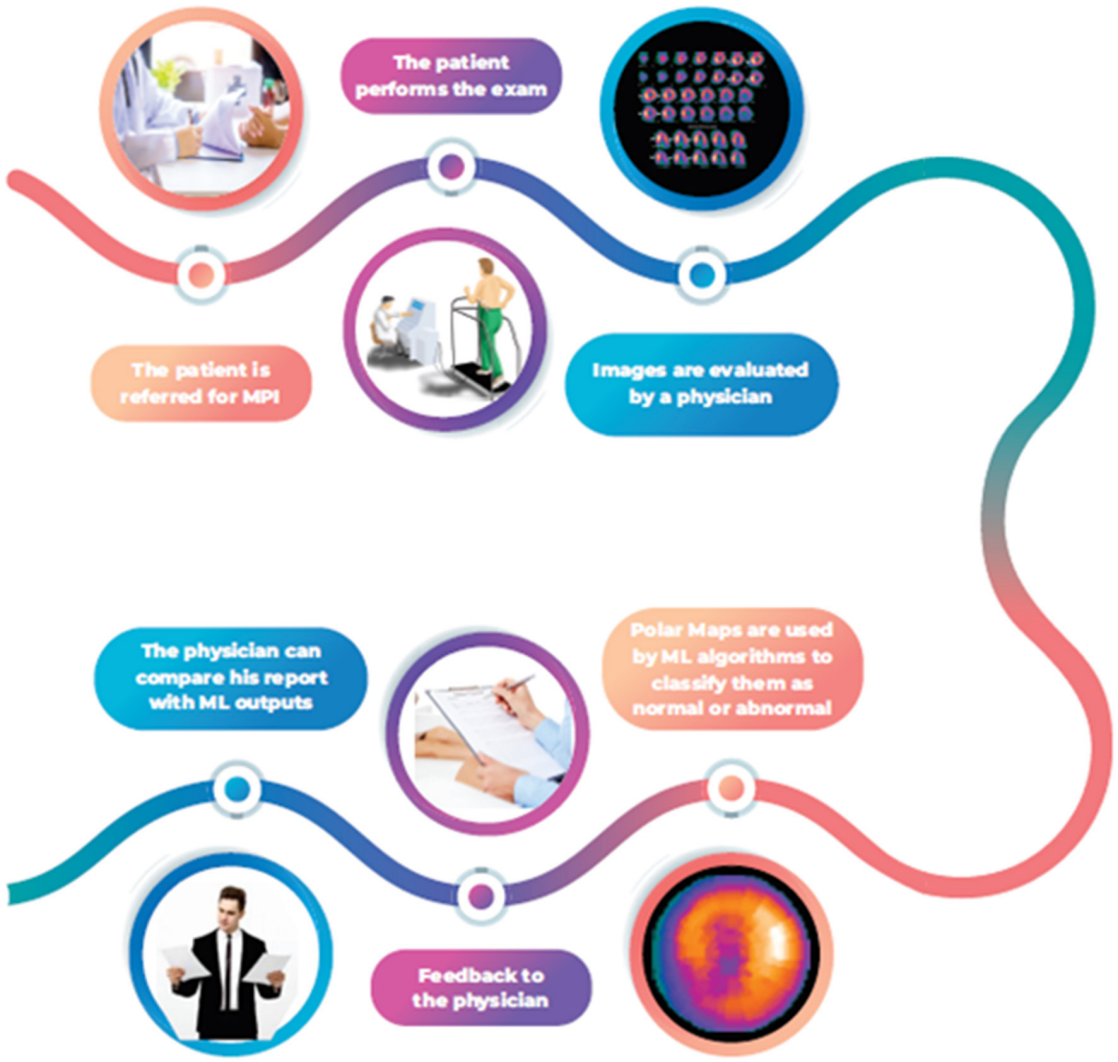
ML algorithms flowchart to support decision making in MPI.

In this context, designing an ML tool to give the physician some support for his MPI reports would be necessary. This tool would be precious for medical residents and other trainees in disagreement between the reader and the algorithm. In case of discordance, in-depth analysis and review will be necessary for the final decision-making, allowing for better comprehension of the method, improved reports' quality, and better training (Figure 1). We evaluated 04 supervised ML algorithms (Adaptive Boosting, Gradient Boosting, Random Forest, and Extreme Gradient Boosting) to distinguish between normal vs. abnormal single-photon emission computed tomography (SPECT) MPI polar maps.

## Materials and Methods

We analyzed 1,007 consecutive MPI studies (January 2016–December 2018). Studies were acquired in the supine position at stress and rest with additional prone imaging at stress for males to correct for diaphragmatic attenuation artifact. All patients underwent an 8-frame ECG-gated 2 day rest-stress Tc-99 m sestamibi myocardial perfusion single-detector SPECT (Millennium MPR, GE Healthcare), according to the ASNC guidelines (10). Rest-stress doses were determined based on the patient's body weight by a factor of 0.25 mCi/kg. Acquisition times were 21 min for stress and rest imaging using a 180° orbit. We reconstructed the transaxial emission images with ordered-subsets expectation maximization (OSEM) algorithm with 04 subsets and 10 iterations and a uniform initial estimate (11). We used Emory Cardiac ToolboxTM (Emory University/ Syntermed, Atlanta, GA) for image reconstruction, axis orientation, and polar maps. MPI studies were analyzed and reported using all the relevant clinical and stress-derived data. We used Emory Cardiac ToolboxTM for image reconstruction, axis orientation, and polar map generation. Images were classified as normal or abnormal using visual analysis, quantitative parameters, and wall motion data. MPI was considered normal in the presence of normal left ventricular cavity size, normal regional wall motion and left ventricular thickening, homogeneous perfusion throughout the myocardium, a normal left ventricular ejection fraction (>45%), and normal right ventricular uptake (12). A single expert reader initially reported these studies, and then a second expert evaluated all the software's polar maps before validating the study. When the studies had conflicting interpretations, the second reader analysis was considered for the ML algorithm. All specialists who participated in the evaluation process have a specialist in nuclear medicine and more than 15 years of experience. The senior researcher responsible for conducting the review and the title of specialist was president of the Brazilian Society of Nuclear Medicine, has over 20 years of experience, and more than 100 articles published in the area. Our work was carried out at Hospital Universitario Antônio Pedro in Brazil, and we only had access to anonymized polar maps in our study. Polar maps were post-processed (GE Healthcare Xeleris®) and exported in.tiff format with matrix size 175 x 175.

So, we generated 02 polar maps (stress/rest) for female patients and 03 (stress/rest/prone) for male patients. We did not use the clinical data for the ML algorithms, but the image attributes such as pixel position and intensity.

In our work, only images from myocardial perfusion were used. The Ethics Committee (Universidade Federal Fluminense) has authorized us to use these images as long as they are anonymized in agreement with the Declaration of Helsinki.

### Features Extraction

**Figure 2:**
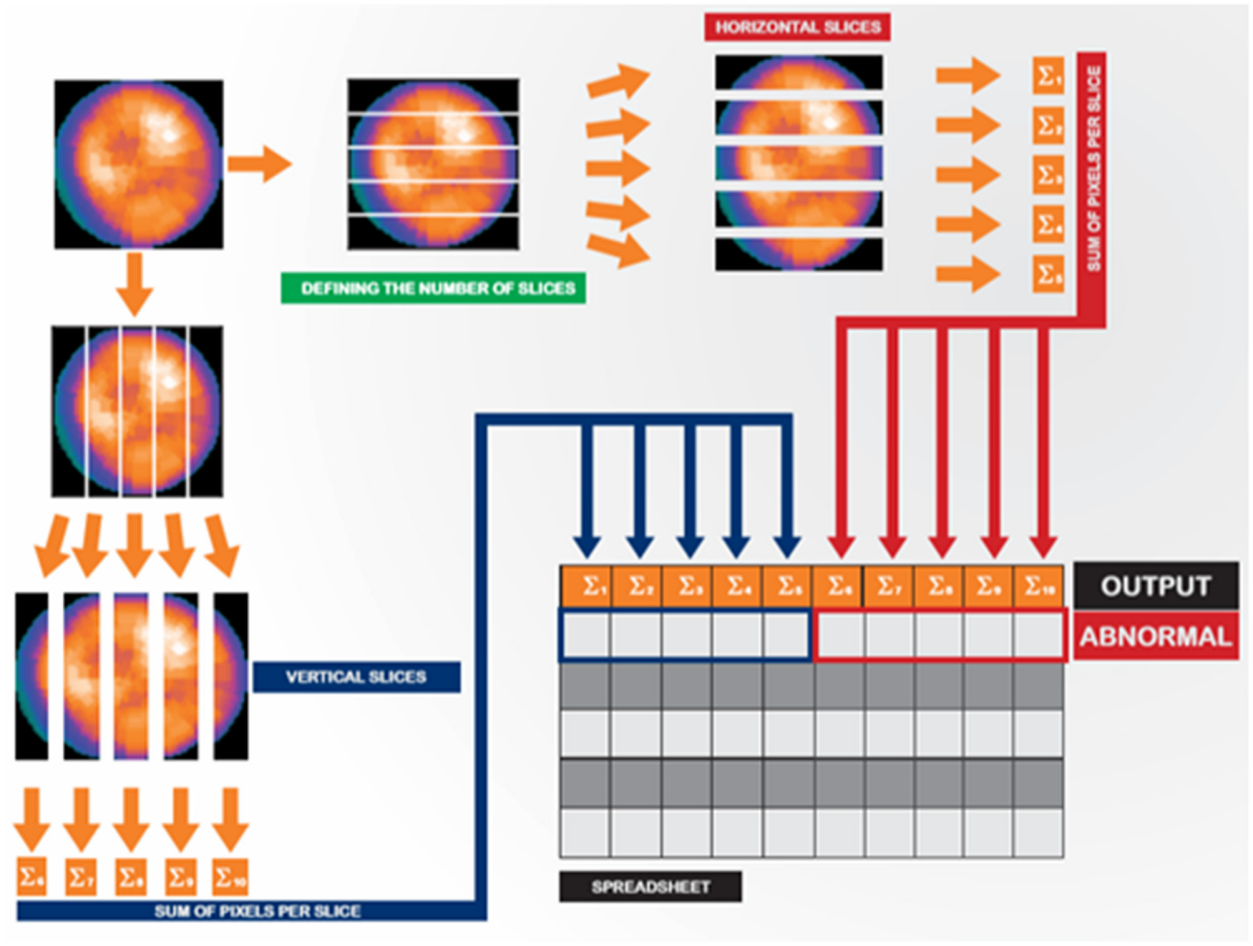
Image slicing and feature extraction strategy.

We extracted image features using an image slicing process based on Ouali et al. (13). In this process, each image was divided into 05 horizontal and 05 vertical slices (Figure 2), where the pixel intensities from each slice were sum, so we obtained a total of 10 attributes. After processing, cardiac nuclear medicine images are traditionally mapped to a colored single-channel representation (the so-called GE color). Thus, we obtained a matrix with 11 columns per 955 rows (one row for each image). The first 10 columns represent the slicing process features, and the last column corresponds to the label indicating whether the polar map is normal (1) or abnormal (0). We complied with the General Data Protection Regulation (GDPR) (14).

### ML Algorithms

Four ensemble ML algorithms were used [Adaptive Boosting (AB), Gradient Boosting (GB), Random Forests (RF), and eXtreme Gradient Boosting (XGB)]. We built a set of base classifiers in ensemble models and classified new data by voting for these classifiers' predictions. RF uses bootstrap aggregation (bagging) in the process of constructing the base classifiers. Boosting techniques (AB, GB, and XGB) were used to properly combine a series of weak classifiers to obtain a stronger one. A weak classifier occurred when a feature's performance was slightly superior to random guessing (15–24). Table A in Section Supplementary Data 1 shows parameters used in our ML algorithms.

We assessed the model's performance using the following metrics: AUC, sensitivity, precision (positive predictive value), and F1 measure (the harmonic mean of the precision and sensitivity). ML algorithms were implemented in Python 3 using open-source libraries (25, 26).

### Cross-Validation

We used ten-fold cross-validation to validate the classification model results (27, 28). In the cross-validation, the training database is divided into k (k = 10) parts of the same size, k-1 of which is for training and testing. Therefore, all data were used in the training process. All images used were independent, and there was no data leak in the cross-validation process.

### Data Augmentation

The database consisted of 108 normal images and 899 abnormal images, an unbalanced training dataset (Table 1). In this context, we increased the number of normal images using the polar maps' geometric properties. This data augmentation was done only in training dataset splits to avoid leakage of information. 324 new polar maps were generated - three for each normal polar map (3^*^108). Therefore, cross-validation was done with 9 boxes containing 202 images in the test set and a training set with the remaining 1,129 images. In the tenth box, the training set consisted of 196 images. Figure A in Section Supplementary Data 2 shows how the process was done. Data used in the models are available in Supplementary Material.

**Table 1:** Polar maps characteristics.

	**Rest M**	**Str M**	**Prone M**	**Rest F**	**Str F**	**Total**
normal	10	10	10	39	39	108
abnormal	91	91	91	313	313	899
Total	101	101	101	352	352	1,007

*Rest M, rest male; Str M, stress male; Prone M, prone male; Rest F, rest female; Str M, stress female*.

## Results

In Table 2, we can see the ML algorithms performance. All models had sensitivity >92%. However, only RF had 96%. AB, GB, and XGB achieved, respectively, 92, 94, 95%. Sensitivity standard deviation was lower in GB, RF, and XGB (0.02). RF had the best accuracy (93%), followed by GB (92%), XGB (92%), and AB (90%). Precision ranged from 0.96 (XGB, RF, AB) to 0.97 (GB) while F1 measure ranged from 0.94 (AB) to 0.96 (RF). All precision standard deviations were 0.01. We achieved the best AUC by RF (0.85), followed by XGB (0.82), GB (0.81), and AB (0.77). AUC standard deviation was lower in GB (0.05). All processing time was lesser than 0.2 s. We obtained the best processing time in AB (0.16).

**Table 2:** Ensemble ML algorithms performance (mean and standard deviation tenfold cross validation results).

**Model**	**Accuracy**	**F1**	**Precision**	**Sensitivity**	**Time (s)**	
**Training set: Mean** **+/- Standard Deviation**						
AB	0.961 +/- 0.006	0.970 +/- 0.004	0.982 +/- 0.004	0.959 +/- 0.005	0.175 +/- 0.023	
GB	0.993 +/- 0.001	0.995 +/- 0.001	0.999 +/- 0.001	0.991 +/- 0.002	0.411 +/- 0.043	
RF	1.000 +/- 0.000	1.000 +/- 0.000	1.000 +/- 0.000	1.000 +/- 0.000	0.297 +/- 0.046	
XGB	1.000 +/- 0.000	1.000 +/- 0.000	1.000 +/- 0.000	1.000 +/- 0.000	0.174 +/- 0.018	
**Model**	**Accuracy**	**F1**	**Precision**	**Sensitivity**	**Time (s)**	**AUC**
**Test set: Mean** **+/- Standard Deviation**						
AB	0.907 +/- 0.032	0.947 +/- 0.020	0.969 +/- 0.014	0.927 +/- 0.044	0.164 +/- 0.044	0.778 +/- 0.068
GB	0.927 +/- 0.020	0.959 +/- 0.012	0.970 +/- 0.012	0.949 +/- 0.027	0.416 +/- 0.056	0.815 +/- 0.059
RF	0.938 +/- 0.017	0.965 +/- 0.010	0.968 +/- 0.015	0.963 +/- 0.025	0.273 +/- 0.048	0.853 +/- 0.070
XGB	0.924 +/- 0.019	0.957 +/- 0.011	0.963 +/- 0.014	0.952 +/- 0.029	0.186 +/- 0.028	0.820 +/- 0.083

*AB, AdaBoost; GB, Gradient Booting; RF, Random Forests; XGB, eXtreme Gradient Boost; AUC, Area Under the Receiver Operating Characteristics Curve (ROC)*.

## Discussion

This study evaluated ML algorithms' ability to distinguish between normal and abnormal SPECT myocardial perfusion polar maps (without specifying the nature of the abnormality). ML algorithms had high accuracy in image classification. Three models obtained AUC higher than 0.80, and had precision and sensitivity > 0.94. The performance was also high if we consider the F1 measure. Indeed, these models can contribute significantly to the decision-making process. The results obtained in this work reiterate the role of these algorithms and their importance in nuclear medicine. They add to other previous successful experiences.

An example of this is Nakajima et al., who used artificial neural networks in CAD diagnosis and had impressive results. The AUC was superior to 0.9 (overall) in all cases tested, including patients with previous myocardial infarction and coronary revascularization (29–32). Another example of successful performance (AUC = 0.81) was the use of an ML algorithm (LogitBoost) to predict early revascularization after myocardial perfusion imaging with SPECT (33, 34). Cortes (35) had good results (AUC = 0.83) using a different ML algorithm, called Support Vector Machine (SVM), to evaluate a patient's risk of cardiac death after adenosine myocardial perfusion SPECT (1).

It is worth mentioning, as pointed out by Elhendy et al. (36), that a study classified as normal has high relevance regarding a patient's prognosis: the annual mortality and cardiac event rate is <1% during 5-year follow-up after a normal MPI. In our application, the best model was RF, although GB and XGB had good results. RF was previously used successfully to predict mental problems in adolescents from specific questionnaires (AUC: 0.739) (37) and to forecast 1-, 2-, 3-, 4- and 5-year all-cause mortality from pre-implant variables of patients submitted to cardiac resynchronization therapy (38), to foresee the outcome of 90Y-radioembolization in patients with intrahepatic tumors (39) and also to predict complete pathological response in rectal cancer after chemoradiotherapy using computed tomography radionics and 18F-fluorodeoxyglucose positron emission tomography (40) and Cantoni et al. (41) have evaluated the performance of SPECT and cadmium-zinc-telluride (CZT)-SPECT in patients with CAD (or suspected) and have compared the diagnostic accuracy using RF. The sensitivity of CZT-SPECT and SPECT were 96 and 88%, respectively. The main advantage of RF is its lower computational cost compared to Deep Learning, for example, generally eliminating Graphics Processing Units (GPUs). RF is a simple and powerful ML algorithm with applications even in other different contexts, such as the prediction of suicidal ideation (42) and right ventricular hypertrophy (43); however, its performance may vary depending, for example, on the application and the parameters used in the ML model. In assessing the first 5-year all-cause mortality separately, the results indicated an AUC that ranged between 0.76 and 0.8, while in predicting the response to chemotherapy, the AUC obtained was 0.94 (38, 40). Also, no ML model is better than the others in all situations. Baskaran and colleagues, for instance, were successful in predicting obstructive coronary artery disease (AUC: 0.779) and revascularization (AUC: 0.958) from clinical and imaging data (44). Thus, it is usually interesting to analyze more than one model. Besides, the increase in the training database can contribute to making these algorithms' performance even better.

Besides the low computational effort for processing the algorithms, another advantage in our study was polar maps. A single two-dimensional image obtained during the stress, rest, or prone phase contains an adequate myocardium representation. However, some distortion may be induced in this process. In light of that, polar maps were used to obtain new images from the rotation (data augmentation) only in the training set, which resulted in a considerable expansion of the training databases in cross-validation. This increase in the database allows for an improved model training process and contributes to better results. It is an important alternative, especially when dealing with a database with an asymmetry of the outcomes-as highlighted by Thabtah and colleagues, a classification algorithm's performance can be affected if the study data is highly unbalanced (45). Kocheturov et al. emphasized that this asymmetry is considered a significant obstacle. It can lead to biased rules in favor of the majority of the result-which requires unique approaches to the issue (46). In our work, for example, images considered normal corresponded to about 10%. However, after carrying out the data augmentation, this value increased to 32%, which significantly improved the imbalance between classes.

Moreover, the storage size of images is small since each image is <25 KB. Another significant advantage is that our slicing process proved to be quite adequate to generate features. The use of vertical and horizontal slicing has already been used in different contexts. Shih et al., for instance, exhibited horizontal and vertical slices taking from 2-day ^99m^Tc-tetrofosmin SPECT images of a patient with duodenogastric reflux in a hiatal hernia (47). Teramoto et al. (48) used these slices to visualize the root system architecture of rice using X-ray computed tomography. Thus, this work brings as a novelty, not the issue of using vertical and horizontal cuts *per se*, but the use of this tool in generating attributes for ML models from polar maps. It was the first time that this type of slicing process was used in nuclear cardiology, to the best of our knowledge. The results obtained in this work suggest a potential use of this type of slicing. However, they do not exclude the possibility or validity of other types of approaches. Betancur et al. also took advantage of using polar maps. They developed successful neural networks (deep learning) to automatically predict obstructive coronary disease from MPI compared with current clinical methods (49). Togo et al. did a similar analysis using PET/CT images, also been successful with the use of polar maps. The idea was to assess the ML model (in case, deep learning) to distinguish between two different outcomes: cardiac sarcoidosis and non-cardiac sarcoidosis (50).

The excellent performance of our tool in the classification of polar maps has mainly two potential applications. The first one would be to analyze polar maps and compare them to the first report originated to assess any potential misinterpretation. Trägardh et al. (51) emphasized that reporting an image is usually the only form of communication between the physician and the caregiver, being one of the critical components of care delivery that can occasionally become legal evidence. Besides, suppose all images obtained during the stress, rest, and prone phase (in the case of a male patient) are considered normal in an ML algorithm evaluation. In that case, the final report should be normal, reducing the risk of mistakes in the medical report with the advantage of only analyzing 2 or 3 images. Also, the physician and ML model will act synergistically, as the model can identify potential errors. The specialist will identify false positives/negatives, which is essential for retraining and improving the models and their performance. Although the costs involved (financial, emotional, and others) could be significant, it seems that there are a few works related to this subject (reviewing previous evaluated reports). We also believe that ML tools could significantly differ in the medical training process in this context. For instance, medical residents can have an additional source to compare their reports, contributing to improving the learning process, therefore supporting their education.

## Limitations and Future Work

There are some limitations to this study: (a) it is essential to point out that we have collected all data retrospectively from a single center. Although the benefits of using ten-fold cross-validation, deriving a predictive model from historical data may affect the generalization of the results since we could have seen changes in the patient's characteristics as time goes by (1, 52). (b) A few experts were responsible for producing the medical report. (c) We used a 2-days protocol. So, we may not be able to use this model for a 1-day protocol. In light of that, it is essential to validate our results in different data and distinct contexts. (d) We did not use any clinical information, which could improve the models' performance. In future work, we believe that the methodology developed here can be applied to other contexts, including polar maps obtained with different radiotracers or even different outcomes, such as the definition of the territory where the abnormality was verified. (e) Possibly the originality of the proposed method could be explained because of our dimensional reduction method. In this context, different types of reduction methods, such as principal component analysis (PCA) (53) and independent component analysis (ICA) (54) could improve our results and should be explored in future work. (f) The model has a focus on predicting only whether the MPI is normal or abnormal.

We did not provide any information on the ischemic heart area and the patient's prognosis. Despite this, we believe that our tool could also help optimize and prioritize reporting queues.

## Conclusions

We have successfully implemented 4 ensemble ML algorithms (RF, GB, XGB, AB) to distinguish normal vs. abnormal SPECT myocardial perfusion polar maps. We used 10 different features extracted using an image slicing process and ten-fold cross-validation. Data augmentation was done in the training set, considering the polar maps' geometric properties and rotating normal images through small angles. The computational times were very low, and RF had the best AUC. We believe that our tool can contribute to a reevaluation of previously reported images and a medical training process for residents, identifying possible mistakes.

## Data Availability Statement

The original contributions presented in the study are included in the article/Supplementary Material, further inquiries can be directed to the corresponding authors.

## Author Contributions

ES, FF, CM, and RG: conception or design of the work. ES, FF, TS, and CM: data collection. ES, FS, and LC: data analysis. ES, FS, CM, RG, CW, and BC: interpretation. ES, FF, CW, BC, FS, CM, and RG: drafting the article. AS and EM: critical revision of the article. ES, FS, CM, and RG: final approval of the version to be published. All authors contributed to the article and approved the submitted version.

## Conflict of Interest

The authors declare that the research was conducted in the absence of any commercial or financial relationships that could be construed as a potential conflict of interest.

## Publisher's Note

All claims expressed in this article are solely those of the authors and do not necessarily represent those of their affiliated organizations, or those of the publisher, the editors and the reviewers. Any product that may be evaluated in this article, or claim that may be made by its manufacturer, is not guaranteed or endorsed by the publisher.
