# Supplementary material for: Machine Learning Algorithms to Distinguish Myocardial Perfusion SPECT Polar Maps
Source: Front Cardiovasc Med. 2021 Nov 11;8:741667. doi: 10.3389/fcvm.2021.741667 (PMC8660123; doi:10.3389/fcvm.2021.741667)
Supplement: Supplementary file 1 [file Data_Sheet_1.docx]

**Supplementary Data 1 (Table A): ML algorithms settings**

|  | **AdaBoost** | **GB** | **XGB** | **RF** |
| --- | --- | --- | --- | --- |
| bootstrap | na | na | na | True |
| ccp_alpha | na | 0.0 | na | 0.0 |
| class_weight | na | na | na | None |
| criterion | na | friedman_mse | na | Gini |
| max_depth | na | 3 | None | None |
| max_features | na | None | na | auto |
| max_leaf_nodes | na | None | na | None |
| max_samples | na | na | na | None |
| min_impurity_decrease | na | 0.0 | na | 0.0 |
| min_impurity_split | na | None | na | None |
| min_samples_leaf | na | 1 | na | 1 |
| min_samples_split | na | 2 | na | 2 |
| min_weight_fraction_leaf | na | 0.0 | na | 0.0 |
| n_estimators | 50 | 100 | 100 | 100 |
| n_jobs | na | na | na | None |
| oob_score | na | na | na | False |
| random_state | None | None | None | None |
| verbose | na | 0 | na | 0 |
| warm_start | na | False | na | False |
| init | na | None | na | na |
| learning_rate | 1.0 | 0.1 | None | na |
| loss | na | deviance | na | na |
| n_iter_no_change | na | None | na | na |
| presort | na | deprecated | na | na |
| subsample | na | 1.0 | None | na |
| tol | na | 0.0001 | na | na |
| validation_fraction | na | 0.1 | na | na |
| algorithm | SAMME.R | na | na | na |
| base_estimator | None | na | na | na |
| base_score | na | na | None | na |
| booster | na | na | None | na |
| colsample_bylevel | na | na | None | na |
| colsample_bynode | na | na | None | na |
| colsample_bytree | na | na | None | na |
| gamma | na | na | None | na |
| gpu_id | na | na | None | na |
| importance_type | na | na | gain | na |
| interaction_constraints | na | na | None | na |
| max_delta_step | na | na | None | na |
| min_child_weight | na | na | None | na |
| missing | na | na | nan | na |
| monotone_constraints | na | na | None | na |
| n_jobs | na | na | None | na |
| **Supplementary Data (Table A): ML algorithms settings (cont.)** | | | | |
|  | **AdaBoost** | **GB** | **XGB** | **RF** |
| objective | na | na | binary:logistic | na |
| reg_alpha | na | na | None | na |
| reg_lambda | na | na | None | na |
| scale_pos_weight | na | na | None | na |
| tree_method | na | na | None | na |
| validate_parameters | na | na | False | na |
| verbosity | na | na | None | na |

na: not applicable. We have implemented all algorithms in Python. Scikit-learn and other libraries also were used.
