# Supplementary material for: Machine Learning Algorithms to Distinguish Myocardial Perfusion SPECT Polar Maps
Source: Front Cardiovasc Med. 2021 Nov 11;8:741667. doi: 10.3389/fcvm.2021.741667 (PMC8660123; doi:10.3389/fcvm.2021.741667)
Supplement: Supplementary file 2 [file Data_Sheet_2.docx]

**Supplementary Data 2 (Figure A): Data augmentation**

The database consisted of unbalanced training dataset of 108 normal images and 899 abnormal images. Acknowledging that an unbalanced database could cause bias, we increased the number of normal images using the polar maps' two geometric properties:

- Rotation of normal polar maps: a polar map considered normal, when rotated through a small angle x (0 <x <4º) both clockwise and counterclockwise, can remain normal. Thus, we ensured that after the rotation, the septum remains thinner than the lateral wall on the left.
- Rotation of abnormal polar maps: a polar map considered abnormal, when rotated through an x-angle, remains abnormal, but the lesions tend to alter the topography. Rotating at a 90º angle, for instance, generates three new images from the original image. Figure A depicted these two properties.

As part of this work, we decided to rotate the normal images through an angle x <4º in the training dataset. An expert (CTM) analyzed and classified all the images generated by rotation. Note that image rotation is a traditional and widely used data augmentation technique. Xue et al., for instance, combined opportunely convolutional neural networks (CNN) and SVM to recognize a type of esophageal microvessel, called intraepithelial papillary capillary loops (IPCLs) - which are intimately related to the depth of tumor invasion of esophageal squamous cell carcinoma. The accuracy obtained was up to 92.74% ^1^. In our work, data augmentation was done only in training tests to avoid leakage of information.

**Figure A: Images Rotation through different angles**
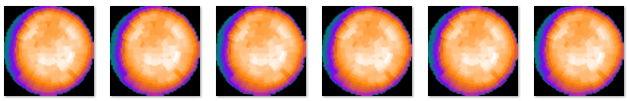


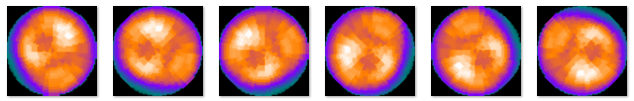


Legend:

1. Rotation through x = 1º, 2º, 357º, 358º, 359º.
2. Rotation through y = 45º, 90º, 135º, 180º, 225º.

**Reference**

1. Xue DX, Zhang R, Feng H, Wang YL. CNN-SVM for Microvascular Morphological Type Recognition with Data Augmentation. [J Med Biol Eng.](https://www.ncbi.nlm.nih.gov/pubmed/28111532) 2016;36(6):755-764.
